# A comparison between Austin-Moore and Corail prosthesis regarding intraoperative periprosthetic femur fractures in hip hemiarthroplasty

**DOI:** 10.1038/s41598-022-10384-9

**Published:** 2022-04-15

**Authors:** Elias Mazzawi, Nabil Ghrayeb, Farouk Khury, Doron Norman, Yaniv Keren

**Affiliations:** 1 Orthopedic Division, Rambam Healthcare Campus, Haifa, Israel; 2 Department of Orthopedic Surgery, University of Ulm, Ulm, Germany

**Keywords:** Fracture repair, Outcomes research

## Abstract

Hip hemiarthroplasty is considered the treatment of choice for displaced femoral neck fractures in elderly less active patients. One important complication of this procedure is an intraoperative periprosthetic femur fracture (IPF), which may lead to poor functional outcome and may increase morbidity and mortality. Our primary aim in this study is to compare between Austin-Moore and Corail prosthesis regarding IPFs. Our secondary aim is to assess patient and surgical technique related risk factors for the development of this complication. Inclusion criteria included patients older than 65 years of age who had a displaced femoral neck fracture and were operated for hip hemiarthroplasty between the years 2014–2018. Patient-specific data was collected retrospectively including age, gender, comorbidities, pre-injury ambulatory status, duration of surgery, surgical approach, use of Austin-Moore or Corail prosthesis, surgeon’s experience and type of anesthesia applied. In addition, radiographs were reviewed for measurement of calcar to canal ratio (CDR) and classification of Dorr canal type. 257 patients with an average age of 83.7 years were enrolled in the study. 118 patients (46%) were treated with an Austin-Moore prosthesis, while 139 (54%) were treated with a Corail prosthesis. A total of 22 patients (8.6%) had intraoperative fractures. Fracture prevalence was significantly higher in the Corail group compared with the Austin-Moore group (12.2% vs. 4.2%, *p* = 0.025). The majority of patients had a Dorr A type femoral canal, while the rest had Dorr B type canal (70% vs. 30%). There was no difference in fracture prevalence between Dorr A and B canal type patients. We didn’t find any significant risk factor for developing an IPF, neither patient wise (age, gender, and comorbidities) nor surgical technique related (surgical approach, type of anesthesia, and surgeon’s experience). Intraoperative periprosthetic fracture prevalence was significantly higher in the Corail patient group compared with the Austin-Moore group. This may be an important advantage of the Austin-Moore prosthesis over the Corail prosthesis.

## Introduction

Femoral neck fractures in the elderly are a major concern. In 2020, there were an estimated 1.5 million hip fractures worldwide^1^, and with the growing aging population this number is expected to rise to 4.5 million by 2050^2,3^. These fractures are associated with a high mortality and morbidity rate, and usually lead to a decrease in daily function including loss of independence^4,5^.

Management of displaced femoral neck fractures can be surgical or conservative. Surgical treatment has been shown to provide lower mortality and morbidity rates, has less complications, and leads to improved rehabilitation when compared to conservative treatment^6–8^. There are various surgical options for treatment of a displaced surgical neck fracture including hemiarthroplasty, total arthroplasty and internal fixation.

The treatment of choice depends on several factors including the patient’s age, general health, and activity level. In addition, it depends on the fracture’s morphology and level of displacement. The NICE fracture guidelines recommend total hip arthroplasty for patients able to walk independently outdoors, who are cognitively competent, medically fit for the procedure, and have a displaced femoral neck fracture. For patients who are less active and older the treatment of choice is hip hemiarthroplasty^9^.

Hemiarthroplasty prosthesis can be divided into monoblock (e.g. Austin-Moore) and modular (e.g. Corail). The latter has a variety of stem sizes depending on the patient’s femoral canal size, with an option for standard vs. high offset neck, as well as different head sizes and lengths, which can fit each patient’s specific anatomy and biomechanics. In monoblock prosthesis, the head, neck and stem are a single piece. Its size usually depends only on the patient’s femoral head size, whereas the neck and stem sizes are usually fixed (some prosthesis have a standard vs. narrow stem). Disadvantages of monoblock prosthesis are less control over the leg length, offset, and less stable fixation in the femoral canal, while its advantages are being a cheaper prosthesis, and possibly a shorter operative time.

One of the important complications of hemiarthroplasties is intraoperative periprosthetic fracture (IPF). This complication increases surgery time, can lead to a poor functional outcome and may increase morbidity and mortality^10^. To date, there are only a few studies assessing this complication in hemiarthroplasty surgery, and to our knowledge there are no studies comparing the rate of this complication between modular hemiarthroplasty and monoblock hemiarthroplasty.

Our primary aim in this study is to assess the risk of IPF in uncemented hip hemiarthroplasty for femoral neck fractures and compare between the rates of IPF for the Corail and Austin-Moore prosthesis. Our secondary aim is to recognize the risk factors for IPF (both patient and surgical technique related).

## Materials and methods

This study included 257 patients who were admitted to our hospital between 2.1.2014 to 29.12.2017, diagnosed with displaced femoral neck fractures and treated with hip hemiarthroplasty.

The patients' medical files along with relevant radiographs were reviewed. Data on age, gender, comorbidities (hypertension, ischemic heart disease, and diabetes mellitus), pre-injury ambulatory status, duration of surgery, surgical approach, use of a Corail or a Austin-Moore prosthesis, surgeon’s experience and type of anesthesia were collected and analyzed.

Inclusion criteria included patients older than 65 years with a displaced femoral neck fracture, who underwent a hip hemiarthroplasty with either a Corail or an Austin-Moore prosthesis. Exclusion criteria included patients with a pathological fracture, a previous ipsilateral hip fracture or a previous ipsilateral hip fixation.

**Figure 1:**
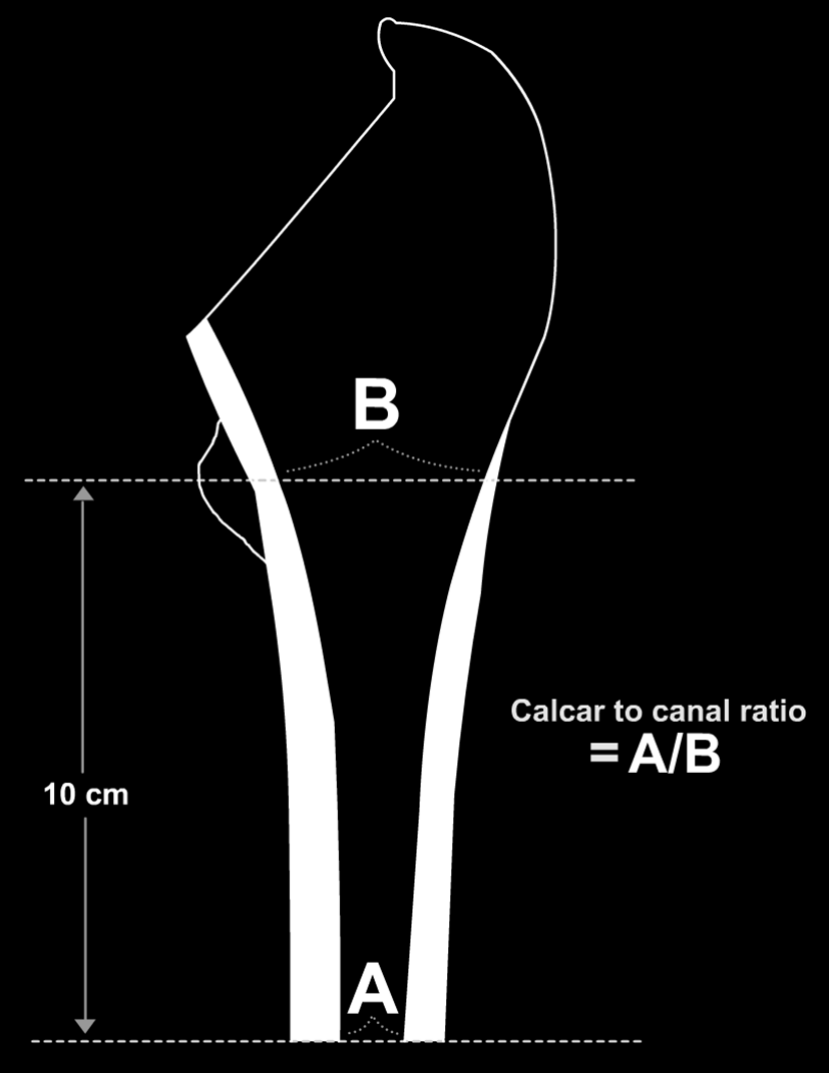
Calcar to canal ratio (CCR) is calculated by dividing the femoral canal diameter at a point 10 cm distal to the mid portion of the lesser trochanter (**A**) by the femoral canal diameter at the mid portion of the lesser trochanter (**B**).

Radiographs were reviewed and analyzed. Considering that Dorr canal type is an established risk factor for IPF^11,12^, calcar to canal ratio (CCR) by Dossick and Dorr^13^, was used to classify femurs. In order to calculate CCR, the femoral canal diameter 10 cm distal to the lesser trochanter was divided by the canal diameter at the mid portion of the lesser trochanter^13^ (Fig. 1).

**Figure 2:**
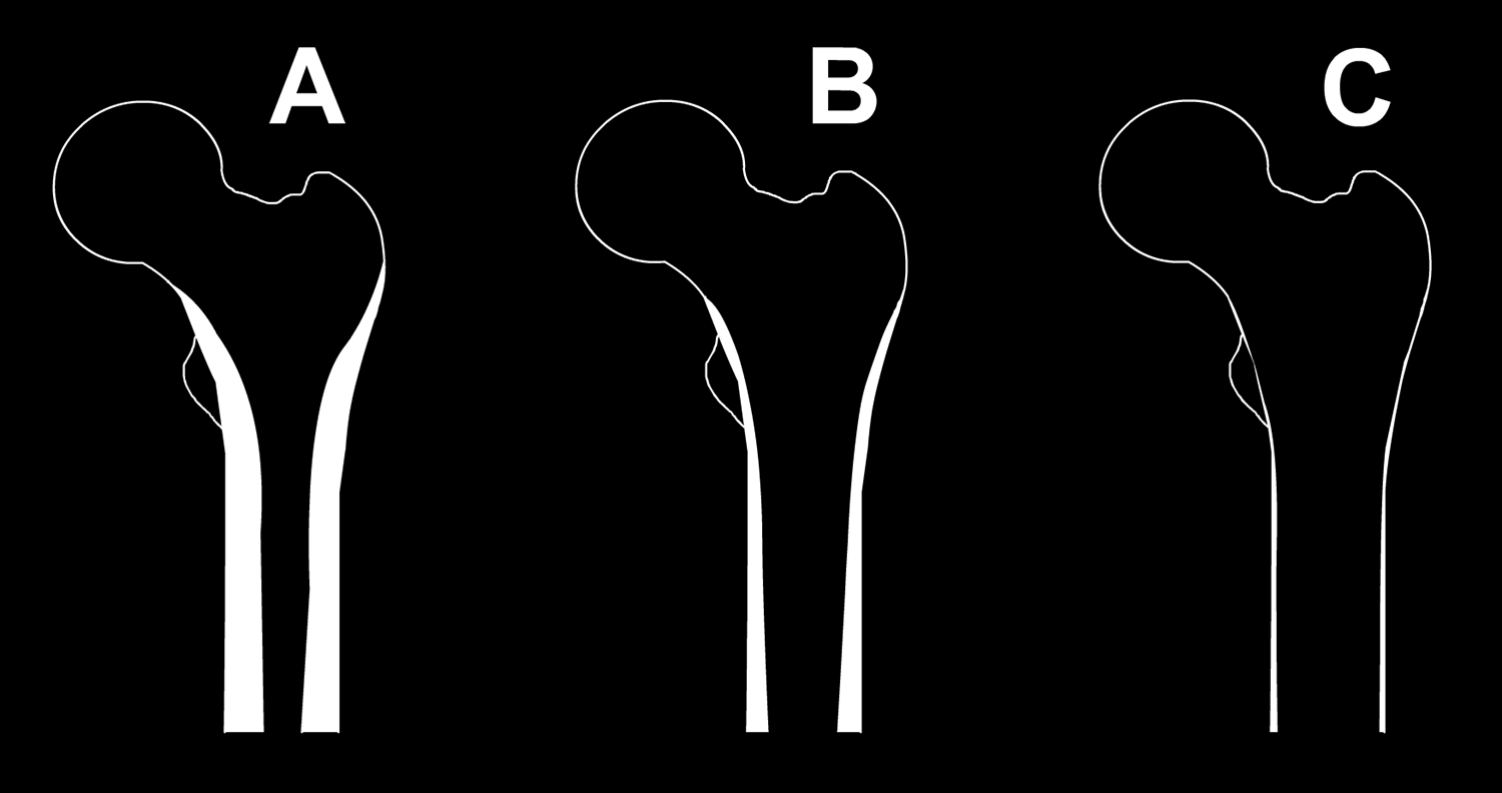
Dorr femur types. Dorr (**A**) represents a femur with thick cortices and narrow canal. Dorr (**B**) represents a femur with thinner cortices and a wider canal. Dorr (**C**) represents a femur with thin cortices and a wide femoral canal.

Lower values Indicate thicker cortices. Dorr A has a CCR of less than 0.5 and represents a femur with thick cortices starting distal to the lesser trochanter which thicken quickly creating a funnel shaped proximal femur. Dorr B has a CCR between 0.5 and 0.75 and represents a wider femoral canal with some bone loss. Dorr C has a CCR of more than 0.75 and indicates considerable bone loss with thin cortices (Fig. 2).

**Figure 3:**
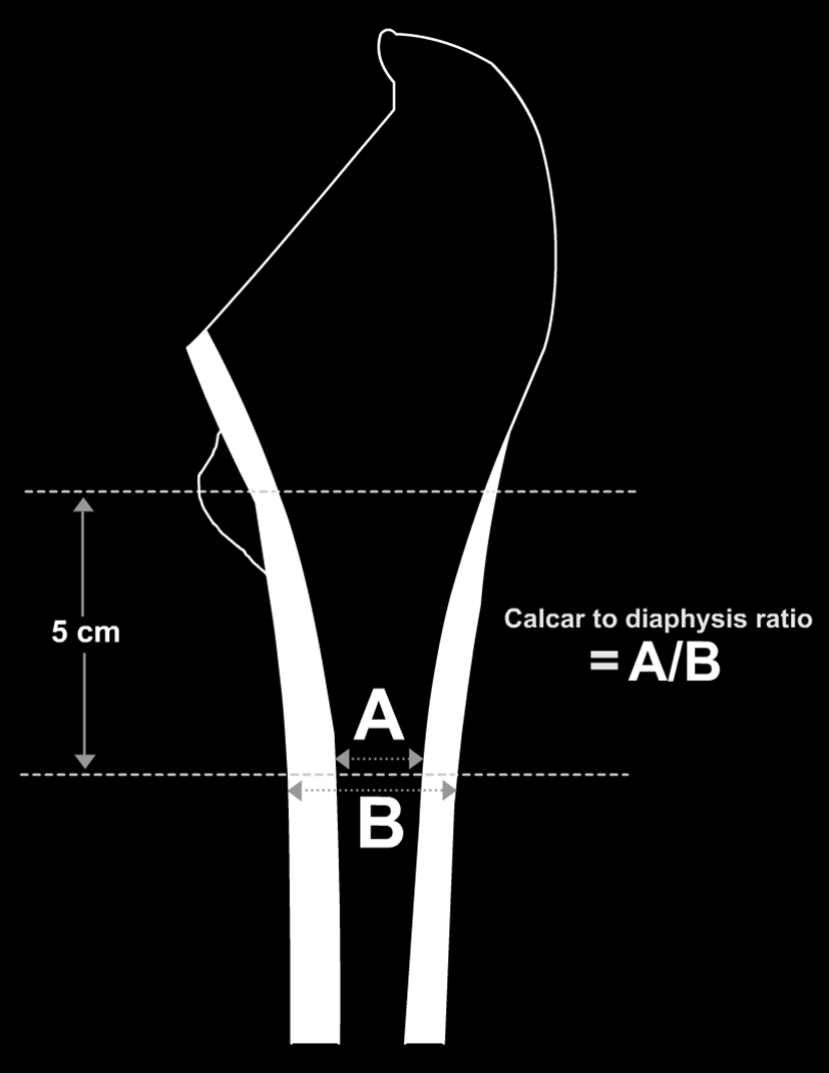
Canal to diaphysis ratio (CDR) is calculated by dividing the femoral canal width at a point 5 cm distal to the mid portion of the lesser (the black arrows) trochanter by the diaphysis width at the same point (the white arrows).

Femurs were also classified using Canal to diaphysis ratio (CDR). CDR was established formerly as a risk evaluation tool for the occurrence of intertrochanteric fractures^14^. CDR was calculated by dividing the femoral canal width by the diaphysis width at a point 5 cm distal from the mid portion of the lesser trochanter (Fig. 3). The higher the ratio, the wider the canal, and hence the thinner the cortex. A CDR more than 0.62 indicates that there is high risk for a hip fracture.

Measurements were taken either on the contralateral femur or on the post-operative ipsilateral femur radiograph due to rotational deformities resulting from the fracture on the admission radiograph.

Preinjury ambulatory status was divided into 4 categories: 1. Freely mobile without aids 2. Mobile outdoors with an aid 3. Mobile mainly indoors 4. Not mobile.

Duration of surgery was recorded in minutes. The three surgical approaches used were the direct lateral (Hardinge), posterior (Moore or Southern) and anterior (Smith-Petersen). Surgical approach was determined mainly by the surgeon's preference.

Hemiarthroplasty surgery was performed using cementless technique. The prosthesis used were either an Austin-Moore prosthesis (Treu Instrumente GmbH, Tuttlingen, Germany) or a collarless Corail Femoral Stem with a Bipolar Head (Depuy International, Leeds, England).

Our institute shifted from using Austin-Moore to Corail prosthesis with a bipolar head for hip hemiarthroplasty in December 2015. This change enabled us to compare between these two prostheses, by comparing between two different time periods—two years before the era of the Corail prosthesis, and two years after. Patients operated between 2.1.2014 and 20.12.2015 were treated with an Austin-Moore prosthesis, whereas patients operated between 26.12.2015 and 29.12.2017 were treated with a Corail prosthesis.

Surgeons were divided into residents and seniors, whereas seniors are surgeons who completed their residency. Types of anesthesia are general, regional which includes spinal, epidural, or peripheral nerve blocks, and combined anesthesia which includes intravenous combined with regional anesthesia.

In case of an IPF, the following information was recorded if available; 1. The anatomical site of the fracture including greater trochanter, calcar, and anterior femoral neck and lateral femoral cortex. 2. The steps leading to the fracture, including: femoral canal broaching and reaming, trial implant insertion and reduction, and final implant insertion and reduction. 3. The IPF treatment utilized including: cerclage wiring, conversion to a cemented stem, and conservative treatment.

Data entry was performed using a spreadsheet application (Excel 2016, Microsoft Corp., Redmond, WA). Frequency tables and descriptive statistics (mean, standard deviation, Min, Max) were presented for all variables. Categorical variables were presented as proportions and continuous variables were presented as mean. Pearson Chi-Square Test and Fisher’s exact test were used for comparison between categorical variables, while Mann–Whitney U test was used for continuous variables. Statistical significance was set at *p* ≤ 0.05 and data analysis was performed using SPSS (SPSS Inc., Chicago, IL, Version 27).

As for the statistical analysis, IPF rates were compared between the Corail and the Austin-Moore groups using Fisher's Exact Test. The univariate analysis tests were used to assess the effects of patient and surgery characteristics on the probability to develop IPF. ROC analysis was performed for CDR and CCR variables to determine the optimal cutoff value and the area under the curve (AUC) was calculated.

All procedures performed in this study were in accordance with the ethical standards of the institutional research committee and with the 1964 Helsinki Declaration and its later amendments or comparable ethical standards. The study was approved by the Bioethics Committee of Rambam Healthcare Campus. Our institutional ethics research committee has determined that there is no need for informed consent, as this is a retrospective study.

## Results

257 patients met the inclusion criteria and were enrolled in the study. The average age of our patients was 83.7. There were 160 (62.3%) females, and 97 (37.7%) males. As for comorbidities 190 (73.9%) patients had hypertension, 67 (26%) had ischemic heart disease, and 69 (26.8%) had diabetes mellitus. In terms of ambulation 30 (11.7%) patients were freely mobile without aids, 61 (23.7%) were mobile outdoors with an aid, 114 (44.4%) were mobile mainly indoors, and 7 patients were not mobile. There was missing data regarding mobility on 45 patients.

The average duration of surgery was 62 min, while the shortest surgery time was 19 min, the longest was 210 min. As for surgical approach, direct lateral was the most popular with 231 (89.9%) patients followed by the posterior approach with 23 (8.9%), and finally the anterior approach with 3 (1.2%) patients. Regarding the prosthesis type 118 (45.9%) patients were treated with an Austin-Moore prosthesis, and 139 (54.1%) were treated with a Corail prosthesis.

Majority of cases 175 (68.1%) were operated by senior surgeons, and 82 cases (31.9%) were operated by residents. In terms of anesthesia, 158 (61.5%) patients were operated under spinal anesthesia, 70 (27.2%) patients were operated under general anesthesia, and the rest were operated under epidural, regional or combined anesthesia. Most patients 180 (70%) had Dorr type A proximal femoral morphology, and 77 (30%) patients had Dorr type B morphology. There were no patients with Dorr type C. As for the CDR classification, there were only 19 (7.4%) patients with a value above 0.62, while the remaining 238 (92.6%) patients had a value equal or below 0.62.

There were 22 (8.6%) patients with an intra operative periprosthetic fracture (IPF) of the femur during hemiarthroplasty for displaced femoral neck fracture. In the Corail group, there were 17 (12.2%) patients out of 139 which had an IPF, while there were 5 (4.2%) patients out of 118 which sustained an IPF in the Austin-Moore group. Anatomical fracture location was recorded in 12 cases out of 22 IPFs, with the anterior femoral neck being the most frequent site (5 cases 41.7%), followed by the calcar with 3 (25%) cases, the greater trochanter, and the lateral femoral cortex with 2 cases each (16.7%).

The steps leading to the fracture were recorded in 20 out of 22 patients with IPF. In 8 (40%) patients the fracture was sustained during final implant insertion, 7 (35%) patients had the fracture during trial insertion and reduction, and 5 (25%) patients sustained the fracture during reaming and broaching. Majority of patients with IPF (20, 90.9%) were treated with cerclage wiring, while one patient was treated conservatively, and another patient was converted to cemented hemiarthroplasty.

There were more IPFs in the Corail group compared to the Austin-Moore group (12.2% vs 4.2%), this difference was statistically significant (*p* = 0.025). We did not find a statistically significant correlation between Dorr canal type (A or B) and sustaining an IPF. Similarly, we did not find a significant correlation between CDR and sustaining an IPF. We assessed patient and surgery characteristics (age, gender, diabetes mellitus, hypertension, ischemic heart disease, surgical approach, anesthesia type, and surgeon’s experience) as individual risk factors for developing an IPF and none of these was found significant. The surgery duration was significantly longer in the group of patients with IPF (*p* < 0.001); However, this can be explained by the additional steps taken for treating the fracture.

## Discussion

IPF is a well-known surgical complication for proximal femoral fractures, as it increases surgery time, can lead to increased morbidity and mortality, and can worsen the functional outcome^10^. Previous studies have compared outcomes of monoblock vs. modular arthroplasty for femoral neck fractures^15–18^. However, we did not find any study comparing IPF rates in modular vs monoblock cementless hemiarthroplasty, which is our first aim in this study. Our secondary aim was to assess the risk factors for developing and IPF in cementless hemiarthroplasty for displaced femoral neck fractures.

The overall incidence of IPF in our study was 8.6% (22 of 257), this finding is consistent with current literature^12,19^. Furthermore, the incidence of IPF in the Corail group was 12.2%, while the incidence of IPF in the Austin-Moore group was 4.2%, this difference is statistically significant (*p* = 0.025).

In order to explain this finding we compared the preparation techniques of both implants. While the Corail prosthesis has reamers and stem sizes varying between 8 and 18, the Austin-Moore has only two reamers and two stem sizes (standard and narrow). This results in versatility, an accurate fit, and a more stable fixation with the Corail prosthesis on the one hand, but on the other hand, reaming in increasing sizes, and fitting a larger stem increases the stress on the cortices and may potentially lead to a fracture. As for the Austin-Moore prosthesis, the stem may also fit loosely in the femoral canal without any stress on the cortices. Thus, the difference between the preparation techniques is probably the most significant factor contributing to development of an IPF.

Furthermore, we compared the designs of the Depuy Corail prosthesis with the Treu Austin-Moore prosthesis. The former has a prominence in the proximal postero-lateral area which requires preparation of the greater trochanter with a box chisel and a rongeur to fit the prosthesis in the medullary canal in neutral position and to avoid unnecessary stress on the cortices which may lead to an IPF. On the one hand an over aggressive preparation may lead to a decrease in bone stock and consequently an IPF, and on the other hand an insufficient preparation may lead to increased stresses on the femoral cortices and result in an IPF. Therefore, it is of critical importance to obtain meticulous preparation to avoid this complication.

Moreover, the Austin-Moore prosthesis has a collared stem which contributes to the stem’s stability inside the femoral canal and can withstand greater forces and loads before subsidence and initiation of a fracture as demonstrated by Whiteside et al.^20^, and by Demey et al.^21^. On the other hand, in our institute we only use collarless Corail stem for hip hemiarthroplasty, hence this may be an advantage of the Austin-Moore over the Corail prosthesis included in our study.

In addition, we should state that we have more experience with the Austin-Moore prosthesis in our institute which we have been using in hemiarthroplasty surgery for years before we switched to the Depuy Corail prosthesis in the end of 2015, and the Corail group in our study included patients operated in the first two years we started using this prosthesis. This may also explain the higher incidence of IPF in the Corail group as there are differences in the surgical techniques between both prostheses, and as expected, a new surgical technique has its learning curve.

We didn’t find any significant risk factor for developing an IPF, neither patient wise nor surgical technique related. These findings are consistent with previous studies^11,22^. As for the Dorr canal type, the patients in our studies were either Dorr Type A or Type B, we did not have any Dorr type C patients. There was not any significant difference in fracture incidence between both Dorr canal type groups. This corresponds to current literature which found a higher fracture incidence only in Dorr type C patients^11,12^, and no significant difference in IPF rates between Dorr A and B. Regarding CDR, we did not find any correlation between this ratio and the incidence of IPF. As stated previously CDR is a tool for evaluating intertrochanteric fractures.

Sustaining an IPF during a hemiarthroplasty for femoral neck fractures is a result of multiple risk factors including femoral bone quality and morphology, and inadequate surgical technique. With the identified risk factors in this study and similar previous studies, the surgeon should take precautions before and during the operation. Preoperative templating is of vast importance as it assesses the stem size and prevents over-sizing which may lead to an IPF. In addition, one should take special precautions in Dorr type C canal type as demonstrated by previous studies^11,12^. Moreover, the surgeon should insist on a meticulous surgical technique suitable for the specific type of prosthesis used. The femoral canal should be prepared adequately without removing too much of the greater trochanter which increases the risk of the IPF, however an insufficient preparation of the greater trochanter may lead to varus position and an IPF because of increased cortical stress. While reaming and inserting the trial/final implant the surgeon should favor low power and consistent blows over aggressive blows^23^. A sudden change in resistance or pitch in this phase is very suspicious for an IPF. An IPF may also be sustained in the trial/final implant reduction phase because of the axial and torsional forces applied on the femur^23^, so the surgeon should be cautious in this phase and consider applying muscle relaxants in cases where considerable force is needed to reduce the joint.

This study has several limitations including its retrospective nature, and the fact that the Corail group included in this study was recorded in the first two years of using the Depuy Corail prosthesis in our institute, which may have influenced the higher fracture incidence in this group, even though we have much experience in hip hemiarthroplasty surgery in our institute and there is not much difference in both surgical techniques. Our main finding was that there were more IPFs in the Corail compared to the Austin-Moore group, and since we didn’t find any similar previous publications, we think that this finding is valuable because it sheds light on a potential advantage of a monoblock prosthesis over a modular prosthesis. Nevertheless, future studies investigating the various discussed points are encouraged.

**Table 1:** Presents an overview of patient demographics and results.

	Austin-Moore group										Corail group								
Age (years)	Maximum			Mean			Minimum				Maximum			Mean			Minimum		
	100			83.9			68				101			83.6			67		
Gender	Male				Female						Male					Female			
	42 (35.6%)				76 (64.4%)						57 (41%)					82 (59%)			
HTN*	98 (83%)										92 (66.2%)								
IHD^+^	36 (30.5%)										31 (22.3%)								
DM^−^	32 (25.4%)										37 (26.6%)								
Preinjury ambulation^$^	1	2			3			4			1		2			3			4
	13 (11%)	21 (17.7%)			62 (52.5%)			6 (0.5%)			17 (12.2%)		40 (28.7%)			52 (37.4%)			1 (0.7%)
Surgery duration (minutes)	Maximum			Median			Minimum				Maximum			Median			Minimum		
	132			57			19				210			58			25		
Surgical approach	Direct lateral			Posterior			Anterior				Direct lateral			Posterior			Anterior		
	103 (87.2%)			15 (12.8%)			0				128 (92%)			8 (5.7%)			3 (0.2%)		
Anesthesia	General			Spinal and Epidural			Combined^#^				General			Spinal and Epidural			Combined		
	32 (27.1%)			86 (72.8%)			0				38 (27.3%)			95 (68.3%)			6 (4.3%)		
DORR	A				B						A					B			
	78 (66.1%)				40 (33.8%)						102 (73.3%)					37 (26.6%)			
CDR	= < 0.62				> 0.62						= < 0.62					> 0.62			
	106 (89.8%)				12 (10.2%)						132 (95%)					7 (5%)			
IPF	Negative				Positive						Negative					Positive			
	113 (95.8%)				5 (4.2%)						122 (87.7%)					17 (12.2%)			
Surgeon’s experience	Senior				Resident						Senior					Resident			
	64 (54.3%)				54 (45.7%)						111 (79.9%)					28 (20.1%)			

*HTN – Hypertension, ^+^IHD – ischemic heart disease, ^−^DM – diabetes mellitus.

^$^Preinjury ambulation 1 = Freely mobile without aids, 2 = Mobile outdoors with an aid, 3 = Mobile mainly indoors, 4 = Not mobile.

Combined Anesthesia includes any combination between general and spinal/epidural/regional anesthesia.

## Data Availability

The datasets generated during and/or analyzed during the current study are available from the corresponding author on reasonable request.
